# CircSNCA downregulation by pramipexole treatment mediates cell apoptosis and autophagy in Parkinson’s disease by targeting miR-7

**DOI:** 10.18632/aging.101466

**Published:** 2018-06-28

**Authors:** Qiuling Sang, Xiaoyang Liu, Libo Wang, Ling Qi, Wenping Sun, Weiyao Wang, Yajuan Sun, Haina Zhang

**Affiliations:** 1 Department of Neurology, China-Japan Union Hospital of Jilin University, Changchun, Jilin 130033, China; 2 Department of Pathophysiology, Jilin Medical University, Jilin, Jilin 132013, China; 3 Department of Rehabilitation, the Second Hospital of Jilin University, Changchun, Jilin 130041, China; * Equal contribution

**Keywords:** pramipexole, Parkinson’s disease, circSNCA, apoptosis, autophagy

## Abstract

We aimed to explore the mechanism of pramipexole (PPX) actions in the treatment of Parkinson’s disease (PD). Genes related to PD and PPX were screened through bioinformatics retrieval. The PD model was constructed by applying 1-methyl-4-phenylpyridinium (MMP+). The RNA expression levels of circSNCA, *SNCA*, apoptosis-related genes (*BCL2*, *CASP3*, *BAX*, *PTEN* and *P53*) and miR-7 were detected by qRT-PCR. Protein expression was determined by western blot. The interactions between circSNCA-miR-7-*SNCA* were verified by dual luciferase assay and immunofluorescence localization. Cell viability was determined by MTT assay. *SNCA* and circSNCA expression levels in PD were downregulated after PPX treatment, consistent with the levels of pro-apoptotic genes. CircSNCA increased *SNCA* expression by downregulating miR-7 in PD as a competitive endogenous RNA (ceRNA). Lower circSNCA expression was associated with the reduced expression of pro-apoptotic (*CASP3*, *BAX*, *PTEN* and *P53*) proteins. CircSNCA downregulation could decrease apoptosis and induce autophagy in PD. In conclusion, the downregulation of circSNCA by PPX treatment reduced cell apoptosis and promoted cell autophagy in PD via a mechanism that served as a miR-7 sponge to upregulate *SNCA*.

## Introduction

Parkinson’s disease (PD) is a progressive neurodegenerative disease that usually presents in people during old or late middle age with noticeable outward symptoms generally appearing in a person’s sixties. The phenotypes of this disorder include progressive deterioration of autonomic and motor functions, with cognitive decline in most cases. Although the underlying etiology of PD is not completely understood, the most common neuroanatomical pathology is the accumulation of misfolded alpha-synuclein (*SNCA*) into intracellular aggregates called Lewy Bodies (LBs), presenting throughout the enteric, peripheral and central nervous systems. Progression of the disease results in the significant loss of the dopaminergic neurons situated in the midbrain substantia nigra pars compacta [1].

Even so, several therapeutic strategies are available to treat the dopamine deficiency of PD and improve motor symptoms. Drugs that slow the progression of dopamine loss are rare, and pramipexole (PPX) is one of them [2]. PPX is a dopamine D2/D3 receptor agonist with proven efficacy in the treatment of PD motor symptoms in early and advanced PD. In studies of cells, rodents and primates, neuroprotective properties that seemed to arise partly via a mitochondria-mediated anti-apoptotic mechanism were shown [2]. Additionally, PDX is a non-ergot dopamine agonist with relatively high *in vitro* specificity and full intrinsic activity at the D2 subfamily of dopamine receptors, with a higher binding affinity to D3 than to D4 or D2 receptor subtypes. PDX can be advantageously administered as a monotherapy or an adjunctive therapy to levodopa to decrease side effects and increase effectiveness in both early and advanced PD treatments [3]. These results were the basis for considering whether there were other mechanisms involved in PPX treatment of PD by regulating gene expression.

Circular RNA (circRNA), consisting of a circular configuration through a typical 5′ to 3′-phosphodiester bond, was recently recognized as a new class of functional molecules. CircRNA consists of no 5′ or 3′ free terminus and is much more stable in cells. The discovery of RNA molecules with circular configurations tracks back to four decades ago [4]. Early studies found some transcripts with non-colinear or shuffled order and implied that these transcripts might be a byproduct of mis-splicing [5]. Later, accumulative evidence consolidated the existence of circular configured RNA molecules such as transcripts of mouse Sry, human ETS1, and DCC [6,7]. Although these pioneer studies have drafted a blueprint for the current circRNA research, the lack of biological functions and comprehensive analysis halted the progression of circRNA research. In the past few years, the advancement of next-generation sequencing technology enabled scientists to perform genome-wide analysis of the expression of circRNAs and to characterize the diverse origins and compositions of circRNAs. In addition, the well-established roles of miRNAs and the theory of competitive endogenous RNA (ceRNA) facilitated the large leap of circRNA research [8]. CircRNAs are abundant in the brain and exosomes, with the capability of traversing the blood–brain barrier [9]. Therefore, they are perfect candidates as potential diagnostic tools for PD.

In our research, we investigated the interactions between PPX and circSNCA to reveal the mechanism of PPX treatment in PD. Additionally, circSNCA was identified as a ceRNA of miR-7 in PD, and its expression was strongly associated with cell apoptosis and autophagy. Our findings provide novel insights into PPX effects and suggest that circSNCA might be a potential target of PD.

## RESULTS

### *SNCA* is related to the mechanism of PPX treatment of PD

**Figure 1:**
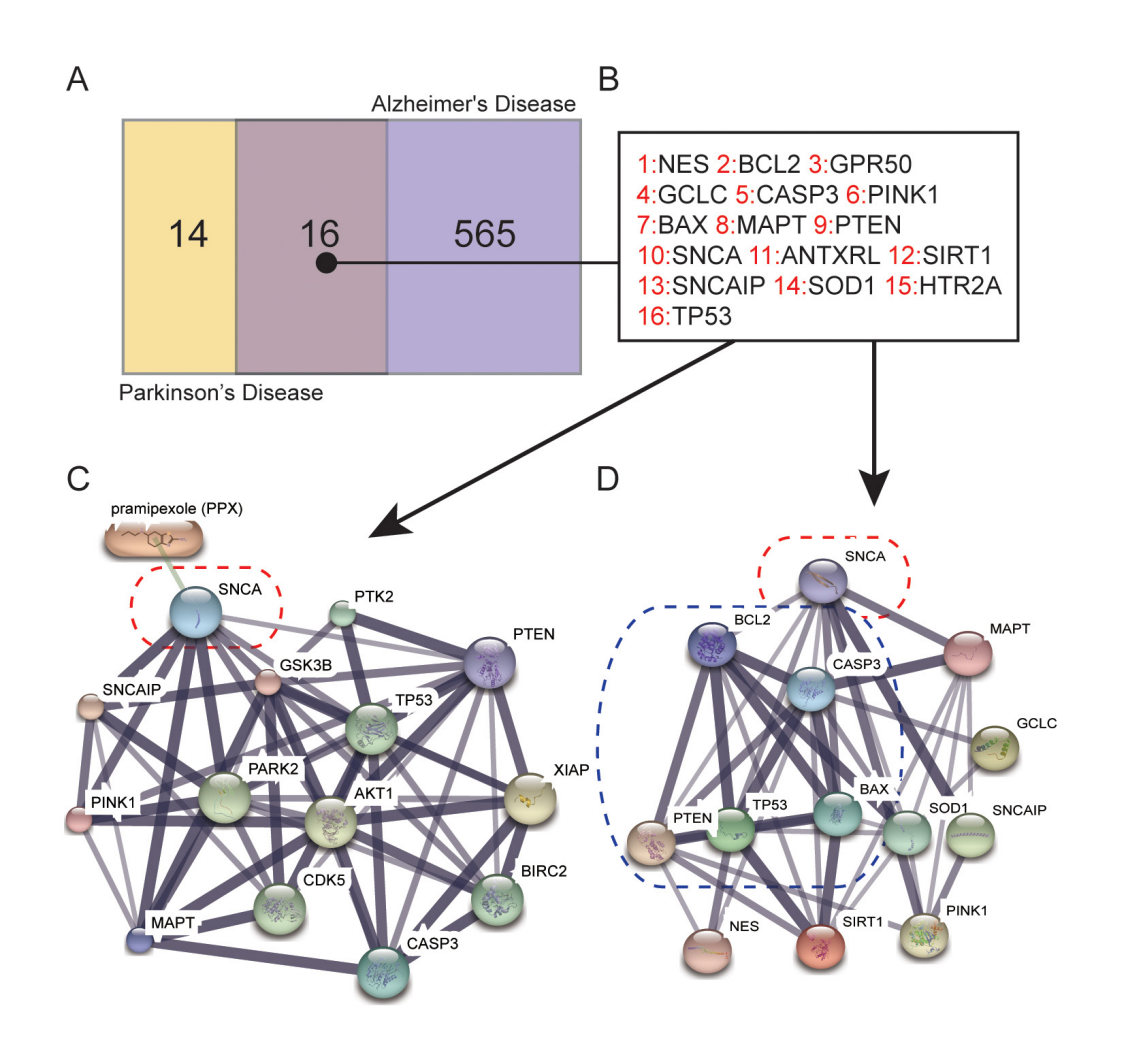
**Genes related to the mechanism of PPX treatment of PD.** (**A**) Number of genes that are concerned solely with PD or AD and with both diseases selected from DiGSeE. (**B**) 16 genes were related to both PD and AD. (**C**) *SNCA* was directly downregulated by PPX according to STITCH. (**D**) The protein-protein interactions (PPI) of apoptotic-related genes with *SNCA*.

There are 30 genes concerned with PD and 581 with Alzheimer’s Disease (AD) [10], among which 16 genes are identical (Figure 1A-B). All of these genes are listed in Table 1. STITCH network analysis [11] was conducted between these 16 genes and PPX (Figure 1C). According to the findings of Wang *et.al* [12] that pramipexole treatment ameliorated SNCA/α-synuclein accumulation, *SNCA* directly responded to PPX treatment in this study. Except for PPX, the PPI network revealed that *SNCA* was closely associated with apoptosis-related genes such as *BCL2*, *CASP3*, *BAX*, *TP53* and *PTEN* (Figure 1D).

**Table 1:** Disease related genes from DiGSeE.

**Parkinson**
MAOB NES BCL2 ATXN2 AIFM1 GPR50 GCLC ATXN3 CASP3 PINK1 BAX MAPT SLC6A3 PTEN PPEF1 CD1D SNCA EPO ANTXRL SIRT1 SNCAIP SOD1 CALY GMCL1P1 CASP1 HTR2A PDYN PDXP COMT TP53
**Alzheimer's Disease**
TSPO SLC1A2 TRPM7 DRD2 CLUL1 HLA-B HIST1H1A SNCG SNCAIP CHRM1 CA2 ERMAP ACTBL2 TBP GAPDH TGM2 CDK5 POMC CTNNBIP1 SLC5A7 NPY CYP46A1 NEFL FPR2 CD4 FGFR2 RTN4R MMP9 NGFR FANCB TTR CNR2 CREBBP SOCS1 RPLP2 NTSR1 TFRC TCF7L2 HSD11B1 RTN3 TLR2 IL4 MPO HTR6 CYP27A1 AQP1 IAPP IGF1R KCNJ4 FGFR3 RELN CTSD MBTPS1 ACE IL1A HLA-C MYC VIM HSPG2 CYP2D6 GKV2D-29 TLR3 CYBB GLUD1 PHF1 COX2 ACAT1 SLC4A1 ADAM10 MKI67 SH2D1A PIK3CA MAPK14 CCND1 ITGAM TFAP2A POLE2 POLE3 SRCRB4D CREB1 GAP43 SHC1 GLUL TERT DYT10 TRAF3IP2 PRRT2 SQSTM1 ADAM17 CAV1 CSF2 SIRT1 KHDRBS1 TARDBP PINK1 PARK2 CSF1 A2M NOTCH1 ETS2 GNRHR F2 HFE TREM2 PREP NFIC CST3 GCLC TP53 CBS CYBA ITGB1 MX1 IFNA1 NR2C1 TMEFF2 SERPINE2 SERPINE1 CCNA2 ENO1 NTS CHKB TIMP1 NCAM1 ATP8A2 ATP5A1 IGKV1-16 CRH TAC1 IL12RB1 IL5RA IL13RA2 IL10RA IL9R DBI PANK2 RYR1 AGTR2 SAR1A AGTR1 GRM2 GRM3 TTPA CD8A CD5 CD7 LRRC15 NPTX1 NRP1 GRN MMP3 MMP12 MMP13 HSPA14 PLA2G7 LAMA1 LAMC1 NRG1 PFKP FAAH HSPA4L RB1 PLTP HSPA1A TRH DBNL CXCL10 P2RX4 MAPK8IP1 PPP2R4 PPA1 KLK6 NMNAT1 WNT1 PDE7A MTFR1 VPS35 SLC30A8 SLC30A1 SLC30A4 SLC30A5 SLC30A6 SLC30A7 WWC1 EFS CALB2 CRYAB ST6GAL2 AGRN IL37 ATG9B NTN1 BRCA2 SNAP25 LTF HMP19 ITGAX LY75 SORL1 PRDX6 PRPF6 TIA1 G3BP1 ZFP36 CHIT1 GAB2 MIR98 MDH2 ADORA2A SESN2 KCNJ10 CD1A CTNNBL1 APLP1 CD80 FGF1 CAMK2G FCGR1A DICER1 SMS LRP8 DBT FGF2 CASP8AP2 DYSF MAPK3 GGA1 EP300 HRAS APBA2 SAA1 LCN2 OLFM1 BLMH PPY FUT3 IGBP1 TSTD1 F11R C9orf3 GNAS ADNP SLCO6A1 SRSF2 HNRNPA1 TRPM2 SRC PIEZO1 ITGB2 APOA2 ALLC MLN PIK3R1 APBA1 CHRNE PTPRC CA9 SYPL1 AATF GRIN2B CLIC1 NAE1 TRHDE MFGE8 APBB1 SHC3 SLC10A3 CHGB FAP CKBE SOCS3 MAP3K1 GRIA1 APPL1 PAWR SDC2 TAP2 ST3GAL4 IGF1 GDNF NUDT6 IGHD2-15 RTN4 MFI2 GRIN1 TFAP4 CREBZF RPP38 LAMP1 TYRP1 FLOT1 CCT LDLR CD44 DISC1 PLD1 LIMK1 CA3 NR3C2 HSH2D RFNG AATK GOSR1 BET1 BECN1 KIF1A NOS1 S1PR5 EGR1 ARG1 PRND BCYRN1 GSK3A VCP ADAMTSL1 EPM2A IFNGR1 ITGA9 ALDH1A1 MIR410 MAML3 MFN2 PADI4 HIST1H1B FOLH1 NEFM TUSC3 GALC PARK7 PDIA3 HNRNPM GCH1 IL12B UBASH3B CDKN1A XPR1 REST HVCN1 HRK COX5A BCL2L1 CD40LG LIF PTN STH RLN1 HTR2A HLA-G DLL1 HES1 RCAN1 PRPH2 PLOD1 GNRH1 IL2 ERN1 CPOX NCKAP1 ND1 SLC39A1 ANTXRL P2RX7 PTEN SERPINF2 ATXN7 APOA1 NMNAT2 NKTR HSP90AA1 HSPD1 PRM1 AZU1 S100A8 RNH1 TGFBR2 SGCG SIX1 SGCA CHRNA4 ROS1 MAOA HBEGF GMFB ATXN8OS C10orf2 KLHL1 PADI3 RUNX3 FMR1 SRA1 EDN1 ATP6V0A4 DHDDS PTH CDK2 TGFB2 TGFB3 CLEC7A PDC DRG1 CSF1R CCNB1 PARP1 CD59 FTSJ2 FTH1 TAS2R62P BPTF ABL1 PLP1 CFLAR ALB GSK3B PRKCD ABCA7 TFCP2 PPP1R10 CDK5R1 ABCA4 TXN MOK PRB1 RFC1 TIMP4 MAP2K4 FCER2 APEH PTGES3 ITGB4 GPI TMSB10 TP73 HNRNPC C1R C4A GRHL3 VIP DLST CRHR1 SND1 IL18R1 HIF1A GSS ERVW-1 CXCL12 DCDC1 NES CRTC1 GEN1 TXNIP CSF3 ALDH2 MCM2 QRFPR MED23 FUS SLC2A3 SNORA62 MMEL1 PHF8 SHH DNMT3A DNMT3B BAG3 LRRK2 PTGER3 XBP1 CSPG4 PRKCA PHB2 PSMD4 CYP19A1 PLP2 PTPRCAP DLG2 DLG4 MAP2 MTNR1A HGF HTT CERK CTH CSE ST7-OT4 COCH

**Figure 2:**
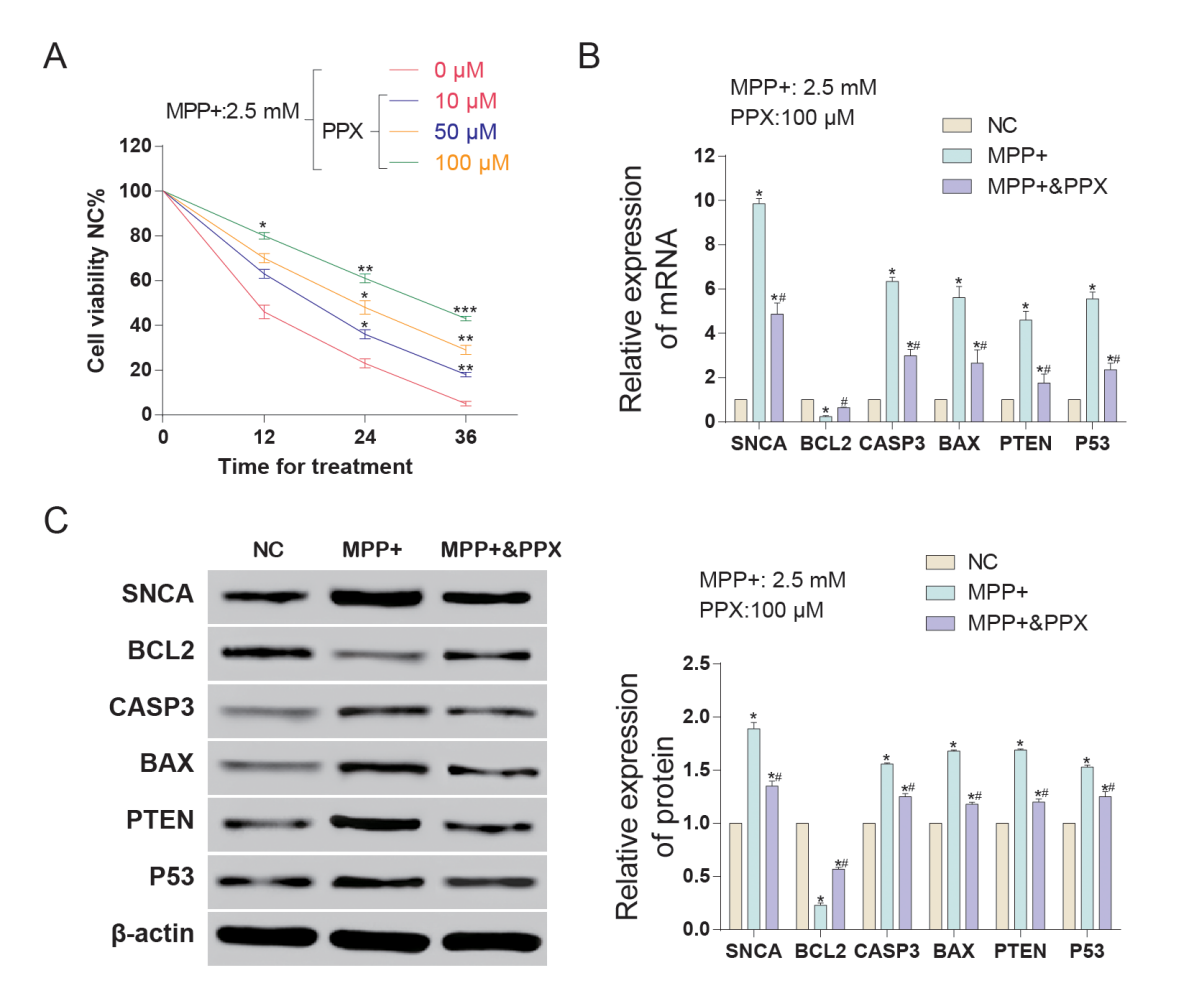
**The expression of *SNCA* and apoptotic-related genes in MMP+ treated SH-SY5Y cells with or without PPX treatment.** (**A**) Cell viability of MMP+ treated SH-SY5Y cells increased with the increase of PPX concentration. *P < 0.05 compared with NC (nothing control), ^#^*P* < 0.05 compared with PD-model (MPP+ 2.5 mM). (**B**) The relative mRNA expression detected by qRT-PCR of *SNCA* and apoptosis-related genes (*BCL2*, *CASP3*, *BAX*, *PTEN* and *P53*). PPX treatment partly offset the influence of MMP+ on the expression of these mRNAs. *P < 0.05 compared with NC (nothing control), ^#^*P* < 0.05 compared with PD-model (2.5 mM). (**C**) The protein expression of *SNCA* and apoptosis-related genes (*BCL2*, *CASP3*, *BAX*, *PTEN* and *P53*) detected by western blot. PPX treatment partly offset the influence of MMP+ on the expression of these proteins. *P < 0.05 compared with NC, ^#^*P* < 0.05 compared with PD-model (MPP+ 2.5 mM).

1-Methyl-4-phenylpyridinium (MPP+)-induced neurotoxicity in SH-SY5Y cells is widely applied as the cell model of PD [13]. After induction of SH-SY5Y cells with 2.5 mM of MPP+ for 12 h, different concentrations (0, 10, 50, or 100 μM) of PPX were added into the mixture for 12, 24 or 36 h. Cell viability was measured by MTT assay, and MPP+ decreased cell viability significantly; however, PPX rescued this situation (Figure 2A). The conditions of 100 μM PPX and 12 h incubation were continually applied in the subsequent experiments (Figure 2B). Predictive genes related to PD were detected by qRT-PCR. *SNCA* shared the same change tendency with *CASP3*, *BAX*, *PTEN* and *P53* (pro-apoptotic genes) but displayed the opposite tendency with *BCL2* (anti-apoptotic gene) (Figure 2B). When treated with MPP+, the *SNCA* mRNA relative expression level was increased sharply compared to the

NC group, while that of cells treated with both MPP+ and PPX was lower but still higher than NC (Figure 2B). The western blot results showed that MPP+ induced increases in *SNCA*, *CASP3*, *BAX*, *PTEN* and *P53* levels and induced a reduction in anti-apoptosis protein *BCL2* (Figure 2C). According to the results, PPX treatment could decrease *SNCA* expression in MPP+-induced PD together with pro-apoptotic genes and increase the expression of anti-apoptotic genes.

### CircSNCA expression was inhibited by PPX

**Figure 3:**
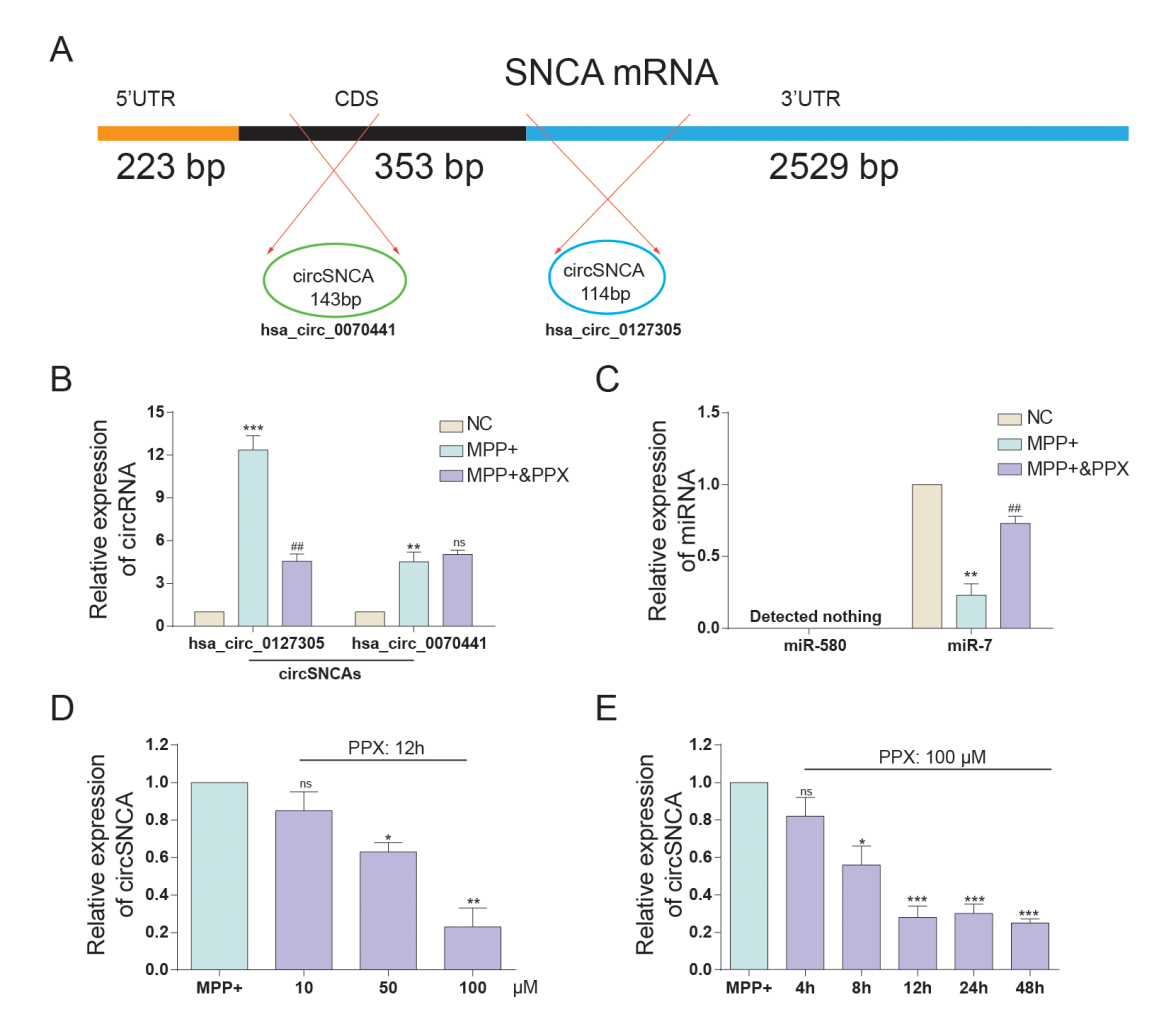
**The expression of circSNCA and miRNA after PPC treatment.** (**A**) *SNCA* mRNA has two corresponding circRNAs, respectively matured from CDS and 3’-UTR. (**B**) The relative expression of hsa_circ_0127305 and hsa_circ_0070441 detected by qRT-PCR increased after PPX treatment. *P < 0.05, compared with NC, ^#^*P* < 0.05 compared with MPP+, and ns meant no significant difference. (**C**) The relative expression of miR-580 and miR-7 detected by qRT-PCR decreased with PPX treatment. *P < 0.05 meant MPP+ compared with NC, ^#^*P* < 0.05 meant MPP+ & PPX compared with MPP+. (**D**) The relative expression of circSNCA detected by qRT-PCR decreased as the concentration of PPX increased. *P < 0.05 compared with MPP+, and ns meant no significant difference. (**E**) The relative expression of circSNCA detected by qRT-PCR decreased as the time for PPX (100 μM) treatment increased. *P < 0.05 compared with MPP+, and ns meant no significant difference.

*SNCA* mRNA and circSNCA are homology-dependent genes. Hsa_circ_0070441 (143 bp) matures from the CDS region of *SNCA* mRNA, and hsa_circ_0127305 (114 bp) matures from the 3’UTR of *SNCA* (Figure 3A). According to qRT-PCR analysis, both had higher expression levels after being treated with MPP+, while the former responded more drastically. When being treated with both MPP+ and PPX, the hsa_circ_0127305 level decreased but was still higher than that of NC. However, there was no significant difference between the MPP+ and MPP+ & PPX groups for hsa_circ_0070441 (Figure 3B). Targeted miRNAs of hsa_circ_0127305, miR-580 and miR-7 were predicted using Circular RNA Interactome algorithm [14]. The relative expression of miR-580 was not detected, and miR-7 was significantly reduced in the MPP+ group compared with NC group. The level of miR-7 was the highest in the MPP+ & PPX group compared with the MPP+ group (Figure 3C). We also tested the circSNCA RNA level under different concentrations of PPX and treatment times (Figure 3D and 3E). The circSNCA level decreased with the increase in the PPX concentration (Figure 3D). For the treatment time, after the first 4 h after PPX treatment, no significant change was evident; however, 8 h after PPX treatment, the circSNCA level decreased, and 12 h after the treatment, the level of circSNCA decreased to the lowest level and remained stable thereafter (Figure 3E).

### Endogenous competition mechanism exists in the circSNCA/miR-7/*SNCA* network

**Figure 4:**
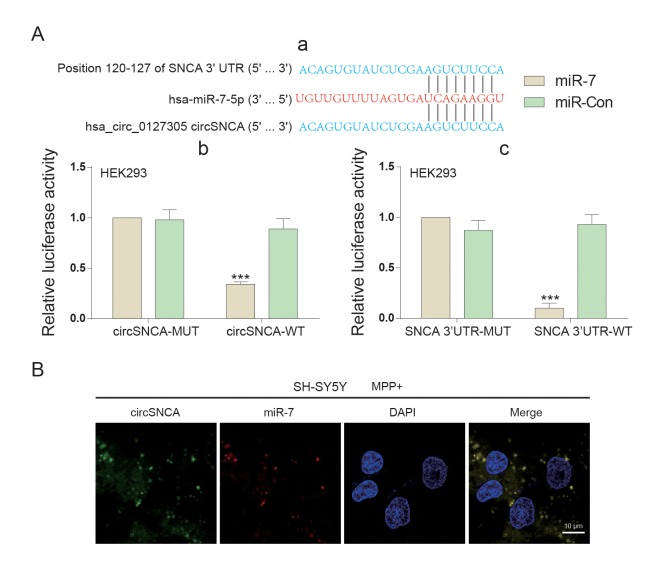
**The target relationship among circSNCA, miR-7 and *SNCA* mRNA of PD.** (**A**) (**a**) 3’-UTR region of *SNCA* mRNA and hsa_circ_0127305 were both found to harbor a binding site for miR-7. (**b**) Luciferase reporter assay results showed that miR-7 exclusively reduced the luciferase activity of the wild-type reporter plasmids of circSNCA. (**c**) Luciferase reporter assay results showed that miR-7 exclusively reduced luciferase activity of the wild-type reporter plasmids of circSNCA. (**B**) RNA FISH for co-localization of circSNCA and miR-7 in cytoplasm of SH-SY5Ys. CircSNCA and miR-7 probes were labeled with Alexa 488 and Cy-5, respectively. Nuclei were stained with DAPI. Scale bar = 10 μm.

**Figure 5:**
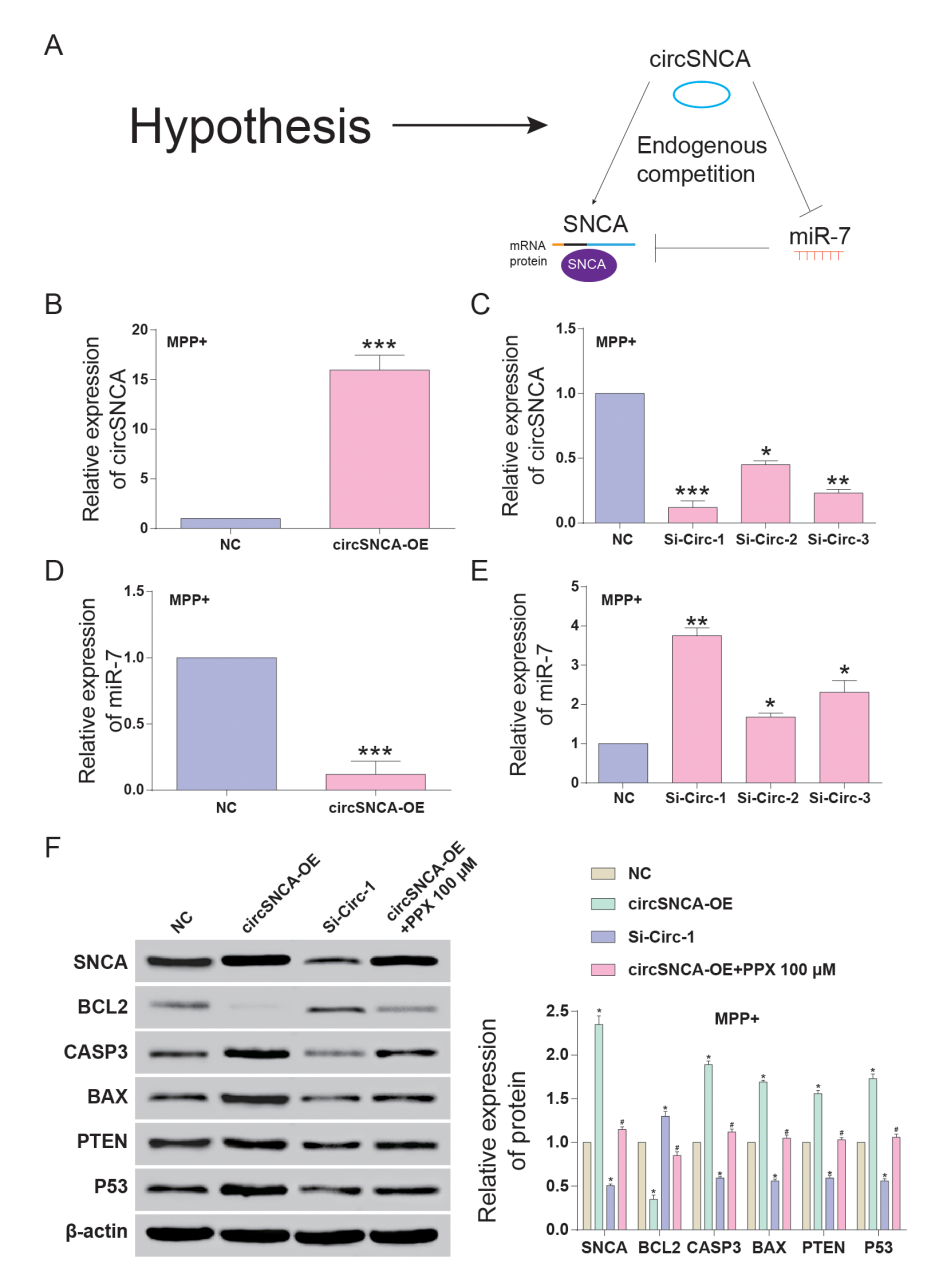
**CircSNCA increased *SNCA* expression by downregulating miR-7 and influenced the expression of apoptosis-related genes.** (**A**) Endogenous competition between circSNCA and *SNCA* mRNA for miR-7 binding. (**B**) The relative expression of circSNCA detected by qRT-PCR increased after overexpression (circSNCA-OE) in the MPP+ group. *P < 0.05 compared with NC. (**C**) The relative expression of circSNCA detected by qRT-PCR decreased after circSNCA knockdown in the MPP+ group. *P < 0.05 compared with NC. (**D**) The relative expression of miR-7 detected by qRT-PCR decreased after circSNCA overexpression in the MPP+ group. *P < 0.05 compared with NC. (**E**) The relative expression of miR-7 detected by qRT-PCR increased after circSNCA knockdown in the MPP+ group. *P < 0.05 compared with NC. (**F**) The protein expression of *SNCA* and pro-apoptotic genes (*CASP3*, *BAX*, *PTEN* and *P53*) detected by western blot increased in the circSNCA overexpression group, decreased in the circSNCA knockdown group, while that of the anti-apoptotic gene (*BCL2*) showed the opposite tendency. *P < 0.05 compared with NC group, ^#^*P* < 0.05 compared with circSNCA-OE.

CircSNCA and *SNCA* 3’UTR had the same target sites of the miR-7 seed region (Figure 4A-a). Double luciferase reporter assays were performed to detect the relationship between circSNCA and miR-7 or miR-7 and *SNCA* mRNA (Figure 4A-b and c). The dual luciferase reporter gene assay results demonstrated that only when circSNCA-WT and *SNCA* 3’UTR–WT were co-transfected with miR-7 was there a sharp reduction in luciferase activity (Figure 4A-b and c). Specific probes for detecting circSNCA (green dots) and miR-7 (red-dots) were transfected into SH-SY5Y cells to evaluate the space sites of circSNCA and miR-7 (Figure 4B). Both green dots and red dots were located in the cytoplasm of SH-SY5Y, with strong space overlap. These two experiments implied that there was endogenous competition between circSNCA and *SNCA* mRNA for miR-7 binding (Figure 5A). To test this hypothesis, we either overexpressed or knocked down circSNCA to investigate how circSNCA regulated miR-7 expression (Figure 5B and 5C). With circSNCA overexpression, the miR-7 level decreased, while with circSNCA knockdown, the miR-7 level increased (Figure 5D and 5E). Compared to the siRNAs (Si-Circ-1, Si-Circ-2, Si-Circ-3) for circSNCA, Si-Circ-1 showed the best effects on circSNCA knockdown. Western blot showed that in the MPP+ group, *SNCA* expression was enhanced with circSNCA overexpression, declined with circSNCA knockdown, and slightly increased with both circSNCA overexpression and PPX treatment, compared to the NC group. Similar results were observed in terms of *CASP3*, *BAX*, *PTEN* and *P53* expression, while opposite results were observed for *BCL2* (Figure 5F). It could be speculated and concluded that PPX had a negative regulation effect on the expression of *SNCA* and pro-apoptotic proteins and a positive regulatory effect on anti-apoptotic proteins. CircSNCA could attenuate the therapeutic effects of PPX in an *in vitro* PD model.

**Figure 6:**
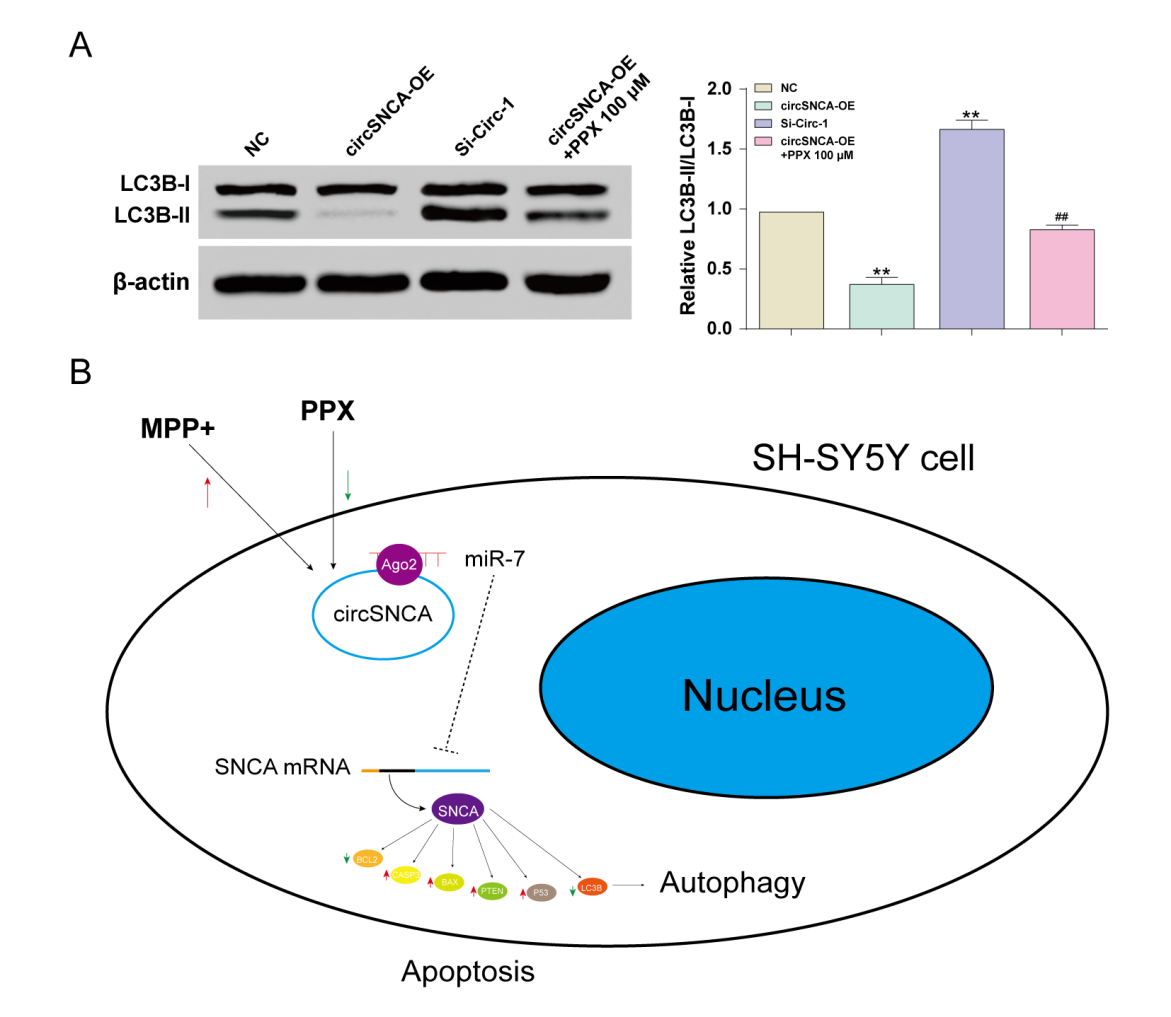
**CircSNCA influenced the expression of autophagy-related proteins.** (**A**) The expression of autophagy-related protein, LC3B-II, detected by western blot decreased in the circSNCA overexpression group, and increased in the circSNCA knockdown group, while that of LC3B-I showed little difference. *P < 0.05 compared with NC group, ^#^*P* < 0.05 compared with circSNCA-OE. (**B**) PPX mediated apoptosis and autophagy of MPP+ treated SH-SY5Y cells by regulating circSNCA, which targeted miR-7.

Furthermore, autophagy-associated protein, LC3B, was also detected by western blot. The LC3B-I level showed no significant change to circSNCA overexpression/ knockdown or PPX treatment. However, the LC3B-II level was low with circSNCA overexpression, high with circSNCA knockdown and slightly low with both circSNCA overexpression and PPX treatment (Figure 6A). To conclude, MPP+ induced an increase in circSNCA in a PD cell model, while PPX reversed it (Figure 6B). The upregulation of circSNCA could sponge and degrade miR-7 through the target sequences and Ago2, which may lead to attenuated inhibition of miR-7 on *SNCA* mRNA and the increased expression of *SNCA*. CircSNCA upregulation also positively correlated with the increasing expression levels of pro-apoptotic proteins (CASP3, BAX, PTEN and P53) and the decreasing levels of anti-apoptotic protein BCL2 and autophagy-associated protein LC3B-II.

## DISCUSSION

In previous studies, the mechanism of the suppressive effect of PPX on PD has not been well understood. In this study, we first identified the significantly reduced expression of *SNCA* and circSNCA after PPX treatment. Furthermore, we investigated the endogenous competition between circSNCA and *SNCA* mRNA and found that circSNCA was a ceRNA of miR-7 in PD, binding with miR-7 and upregulating its target gene, *SNCA*. Additionally, the expression of pro-apoptotic genes (*CASP3*, *BAX*, *PTEN* and *P53*) was reduced, while that of anti-apoptotic protein BCL2 and autophagy-related protein LC3B-II was increased with the downregulation of circSNCA, revealing the inhibition of apoptosis and the promotion of autophagy in PD.

Since circRNAs were newly identified as players in the regulation of post-transcriptional gene expression, studies on their effects on PD have been limited. Interacting with disease-associated miRNAs is one of the important mechanisms of circRNA involvement in disease progression [15]. Multiple previous studies have discussed the function of circRNAs as a sponge of miRNAs to influence pathological processes [16]. In PD, circRNA zip-2 knockdown can lead to the reduced aggregation of *SNCA* protein by sponging miR-60, thus leading to better survival outcomes of PD patients [17]. By sponging miR-7, circRNA s-7 can promote vital genes associated with PD and AD [18]. In our study, we also identified a similar mechanism of circSNCA, which acted as a ceRNA of miR-7 and upregulated *SNCA* in PD. In addition, we found that circSNCA expression was closely related to PPX treatment. It could be speculated that PPX treatment attenuated the progression of PD partly due to its suppressive effects on circSNCA expression.

CircSNCA’s function in PD was revealed for the first time in this study. However, the effect of circSNCA strongly relied on its direct and indirect regulation on miR-7 and *SNCA*, respectively. MiR-7 was believed to be closely coupled to ciRS-7, and the fine-tuning of the miR-7/miR-671/ciRS-7 axis likely plays profound roles in human cancer development [19]. MiR-7 was reported to bind the 3’ UTR of *SNCA* and inhibited its translation, which was confirmed in our study [20]. Tarale *et al.* proved that the low level of miR-7 implied a higher risk of idiopathic PD [21]. Zhou *et al.* suggested that miR-7 inhibited neuroinflammation in the pathogenesis of PD through targeting Nod-like receptors [22]. Li *et al.* demonstrated that miR-7 exerted inhibitory effects on neuronal apoptosis of PD by targeting *BAX* and *Sirt2* [23]. In this specific case of PD, circSNCA facilitated the pathological processes as a miR-7 inhibitor, further verifying that miR-7 was a suppressive player for PD.

*SNCA* is of great importance in the occurrence and development of PD as accumulated evidence has proved its association with this disease. With some correlation experiments, SNCA was found related to neurotoxicity and the anti-apoptosis pathway [24]. When the extracellular environment is broken, the unbalance of gene expression appeared in neurocytes, such as an abnormal level of SNCA, followed by the changes of cell autophagy and cell apoptosis [25]. Maybe in the following process, the nerve cell damage and apoptosis in turn accelerated SNCA expression (anti-apoptosis) [26]. The rapid expression causes misfolding and aggregation of alpha-synuclein, one of the typical features of Parkinson’s [27]. Abnormal *SNCA* aggregation in LBs has been suggested as one of the main causes for PD, which is related to a deficiency in the ubiquitin-proteasome system and the autophagy-lysosomal pathway [28]. It was reported to be closely connected with cell apoptosis and autophagy [29]. During neuronal apoptosis, the aggregation of *SNCA* was realized by histones [26]. Its toxicity was partly due to the defects of autophagy-mediated clearance, and autophagy mediated by transcription factor EB could rescue the midbrain dopamine neurons from *SNCA* toxicity [30]. In this study, we also studied some apoptosis- and autophagy-related genes and found that apoptosis was reduced while autophagy was promoted with the downregulation of *SNCA*, which could help slow down the deterioration of PD. Since *SNCA* downregulation resulted from circSNCA knockdown, it could prove that circSNCA inhibition was effective in PD treatment.

Some limitations existed in this study. For instance, only cell experiments were conducted, and animal experiments must be carried out to prove this mechanism. Additionally, the mechanism itself should be explored more deeply and thoroughly, and some details are still not clear.

In summary, we verified that PPX treatment for PD could downregulate circSNCA. Since circSNCA served as a ceRNA that sponged miR-7 and upregulated *SNCA*, its downregulation by PPX treatment could reduce the expression of *SNCA*. The inhibition of circSNCA and *SNCA* reduced apoptosis and promoted the autophagy of SH-SY5Y cells, attenuating the progression of PD.

## MATERIALS AND METHODS

### Bioinformatics retrieval

DiGSeE (http://210.107.182.61/geneSearch/) is a search platform for genetic bases of human diseases. “Parkinson” and “Alzheimer’s Disease” were used as keywords during co-existing gene selection. The interactions between these genes and PPX were determined and plotted via STITCH (http://stitch.embl.de/) and protein-protein interactions were analyzed on STRING (https://string-db.org/), with the calculation performed by Dijkstra algorithm.

### Reagents and antibodies

PPX was purchased from Tocris Bioscience. MPP+ was purchased from Sigma (St. Louis, MO, USA). The primary antibodies for immunoblot analysis are listed as follows: *SNCA* (CST, Danvers, MA, USA, 2642), LC3BI/II (Abcam, ab51520), *CASP3* (Abcam, ab2302), *BAX* (Abcam, ab32503), *PTEN* (Abcam, ab32199), *P53* (Abcam, ab1431), *BCL2* (Abcam, ab32124) and β-actin (Abcam, ab8227). SiRNAs for knockdown of circSNCA were synthesized by GenePharma company (Shanghai, China). pLCDH-ciR (GeneSeed, China) was used to overexpress circSNCA. FITC-labeled circSNCA probe and Cy3-labeled miR-7 probe were synthesized by Sangon Biotech. The transfection reagent utilized was Lipofectamine 2000 (Invitrogen, Shanghai, China).

### Cell lines and cell culture

SH-SY5Y cells (BNCC338056, BeNa Culture Collection, Beijing, China) were grown in high-glucose Dulbecco’s-modified eagle medium (DMEM-H) with 10% fetal bovine serum (FBS) containing glutamine and sodium pyruvate, in a 5% CO_2_ humidified incubator at 37°C.

### MTT assay

After 12 h treatment of 2.5 mM MPP+ with 10, 50, or 100 μM PPX, the viability of SH-SY5Y cells was identified via MTT assay. 1 mg MTT was added to each milliliter of medium and incubated at 37°C for 4 h. After 4 h, the medium in the plate was discarded, and in each well, 200 μL dimethyl sulfoxide (DMSO) was added before 1-min shaking for dissolution in a microplate reader (Bio-Rad Model 680; Bio-Rad, Hercules, CA, USA). The absorbance of cells in each well was measured at 570 nm, and the cell growth curve was drawn based on an average of five wells. The experiment was repeated in triplicate.

### Western blot

SH-SY5Y cells were lysed in radio-immunoprecipitation assay (RIPA) buffer (BioVision, Milpitas, CA, USA). Total protein in supernatants was quantified using the BCA-200 protein assay kit (Pierce Biotechnology, Rockford, IL, USA). The protein was separated with 12% sodium dodecyl sulfate polyacrylamide gel electrophoresis (SDS-PAGE) and transferred onto a polyvinylidene fluoride (PVDF) membrane (500 mA). The membrane was sealed in Tris Buffered Saline Tween (TBST) with 5% skim milk at room temperature for 1 h, and subsequently incubated with primary antibodies at 4°C overnight. After TBST-washing three times, the membrane was incubated for 1 h at room temperature with secondary antibody. Protein bands were visualized using enhanced chemiluminescence (Santa Cruz Biotechnology; Santa Cruz, CA, USA). Quantity One software (Bio-Rad) was used for image analysis. The results were analyzed by Image-Pro Plus 5.0 (Media, Cybernetics, USA). β-actin was included as the internal control.

### Reverse transcription and quantitative PCR (qRT-PCR)

The total RNA was extracted using Trizol (Invitrogen, Carlsbad, CA, USA). The obtained RNA was reverse transcribed to cDNA using RevertAid First Strand cDNA Synthesis Kit (Thermo, Shanghai, China), and Power SYBR Green PCR Master Mix (Thermo, Shanghai, China) was used for determination. PCR program: predenaturation at 95^o^C for 10 min, followed by 40 cycles of denaturation at 95^o^C for 15 s, annealing at 60^o^C for 30 s, and extension at 72^o^C for 30 s. Subsequently, the dissolution curve of PCR products was generated. With GAPDH expression as the standard for mRNA and U6 expression as the standard for miRNA, relative mRNA and miRNA expression was calculated by the 2-^ΔΔCt^ method. The primer sequences are supplied in Table 2.

**Table 2:** QRT-PCR primers and circSNCA siRNAs.

**Gene**	**Farword(5'→3')**	**Reverse(5'→3')**
SNCA	CCTCAGCCCAGAGCCTTTC	CCTCTGCCACACCCTGCTT
BCL2	GAGGATTGTGGCCTTCTTTG	CGTTATCCTGGATCCAGGTG
CASP3	TCTGGTTTTCGGTGGGTGTG	CGCTTCCATGTATGATCTTTGGTTC
BAX	GTGAGCGGCTGCTTGTCTGG	CTTCCAGATGGTGAGCGAGG
PTEN	GGAAAGGGACGGACTGGTGT	GACTGGGAATTGTGACTCCC
P53	GTCGGACAAGCGGCAGATTG	CCTTCGTCTTAGGGTGAGGC
circ-0127305	CCATCAGCAGTGATTGAAATCTG	ACTGGGCACATTGGAACTGA
circ-0070441	AGAAGACAGTGGAGGGAGCA	GGCTACTGCTGTCACACCC
GAPDH	TCGGAGTCAACGGATTTGGT	TTCCCGTTCTCAGCCTTGAC
miR-580	GCGCTTGAGAATGATGAATC	GAATACCTCGGACCCTGC
miR-7	GCCTGGAAGACTAGTGATTT	GAATACCTCGGACCCTGC
U6	CTCGCTTCGGCAGCACA	AACGCTTCACGAATTTGCGT
**has_circ_0127305 siRNAs (5'→3')**		
Si-circ-1	GATTGAAATCTGCTGACAGAT	
Si-circ-2	AGTGATTGAAATCTGCTGACA	
Si-circ-3	GCAGTGATTGAAATCTGCTGA	

### Dual luciferase reporter gene assay

Luciferase reporter gene recombinant plasmids were inserted with the sequences of wild-type (WT) and mutated type (MUT) circSCNA, WT and MUTSCNA 3’-untranslated region (3’-UTR). MiR-7 mimics or control were co-transfected with WT and MUT circSCNA into the 293 cell line (BeNa Culture Collection, Beijing, China) using Lipofectamine 2000 (Invitrogen). Luciferase Dual Assay Kit (Thermo Fisher Scientific) was used for dual-luciferase reporter gene assay 48 h after cells were transfected.

### Immunofluorescent localization

3×10^4^ SH-SY5Y cells were plated onto slides for 24 h of growth in advance of the probe transfection experiments. 20 nM probes of FITC-labeled circSNCA or Cy3-labeled miR-7 (Sangon Biotech) were co-transfected into SH-SY5Y cells for 36 h. After culture incubation, the cells were digested by trypsin and fixed onto slides. The nuclei were stained with DAPI, and the images were collected using fluorescence microscopy (Carl Zeiss, Jena, Germany).

## References

[1] Smeyne M, Smeyne RJ. Glutathione metabolism and Parkinson’s disease. Free Radic Biol Med. 2013; 62:13–25. 10.1016/j.freeradbiomed.2013.05.001 23665395 PMC3736736

[2] Schapira AH, McDermott MP, Barone P, Comella CL, Albrecht S, Hsu HH, Massey DH, Mizuno Y, Poewe W, Rascol O, Marek K. Pramipexole in patients with early Parkinson’s disease (PROUD): a randomised delayed-start trial. Lancet Neurol. 2013; 12:747–55. 10.1016/S1474-4422(13)70117-0 23726851 PMC3714436

[3] Silindir M, Ozer AY. The benefits of pramipexole selection in the treatment of Parkinson’s disease. Neurol Sci. 2014; 35:1505–11. 10.1007/s10072-014-1891-5 25038745

[4] Hsu MT, Coca-Prados M. Electron microscopic evidence for the circular form of RNA in the cytoplasm of eukaryotic cells. Nature. 1979; 280:339–40. 10.1038/280339a0 460409

[5] Cocquerelle C, Mascrez B, Hétuin D, Bailleul B. Mis-splicing yields circular RNA molecules. FASEB J. 1993; 7:155–60. 10.1096/fasebj.7.1.7678559 7678559

[6] Capel B, Swain A, Nicolis S, Hacker A, Walter M, Koopman P, Goodfellow P, Lovell-Badge R. Circular transcripts of the testis-determining gene Sry in adult mouse testis. Cell. 1993; 73:1019–30. 10.1016/0092-8674(93)90279-Y 7684656

[7] Cocquerelle C, Daubersies P, Majérus MA, Kerckaert JP, Bailleul B. Splicing with inverted order of exons occurs proximal to large introns. EMBO J. 1992; 11:1095–98. 1339341 10.1002/j.1460-2075.1992.tb05148.x PMC556550

[8] Hsiao KY, Sun HS, Tsai SJ. Circular RNA - New member of noncoding RNA with novel functions. Exp Biol Med (Maywood). 2017; 242:1136–41. 10.1177/1535370217708978 28485684 PMC5478007

[9] Li Y, Zheng Q, Bao C, Li S, Guo W, Zhao J, Chen D, Gu J, He X, Huang S. Circular RNA is enriched and stable in exosomes: a promising biomarker for cancer diagnosis. Cell Res. 2015; 25:981–84. 10.1038/cr.2015.82 26138677 PMC4528056

[10] Kim J, So S, Lee HJ, Park JC, Kim JJ, Lee H. DigSee: disease gene search engine with evidence sentences (version cancer). Nucleic Acids Res. 2013; 41:W510-7. 10.1093/nar/gkt531 23761452 PMC3692119

[11] Szklarczyk D, Santos A, von Mering C, Jensen LJ, Bork P, Kuhn M. STITCH 5: augmenting protein-chemical interaction networks with tissue and affinity data. Nucleic Acids Res. 2016; 44:D380–84. 10.1093/nar/gkv1277 26590256 PMC4702904

[12] Wang JD, Cao YL, Li Q, Yang YP, Jin M, Chen D, Wang F, Wang GH, Qin ZH, Hu LF, Liu CF. A pivotal role of FOS-mediated BECN1/Beclin 1 upregulation in dopamine D2 and D3 receptor agonist-induced autophagy activation. Autophagy. 2015; 11:2057–73. 10.1080/15548627.2015.1100930 26649942 PMC4824582

[13] Kim MJ, Park M, Kim DW, Shin MJ, Son O, Jo HS, Yeo HJ, Cho SB, Park JH, Lee CH, Kim DS, Kwon OS, Kim J, et al. Transduced PEP-1-PON1 proteins regulate microglial activation and dopaminergic neuronal death in a Parkinson’s disease model. Biomaterials. 2015; 64:45–56. 10.1016/j.biomaterials.2015.06.015 26117230

[14] Dudekula DB, Panda AC, Grammatikakis I, De S, Abdelmohsen K, Gorospe M. CircInteractome: A web tool for exploring circular RNAs and their interacting proteins and microRNAs. RNA Biol. 2016; 13:34–42. 10.1080/15476286.2015.1128065 26669964 PMC4829301

[15] Ghosal S, Das S, Sen R, Basak P, Chakrabarti J. Circ2Traits: a comprehensive database for circular RNA potentially associated with disease and traits. Front Genet. 2013; 4:283. 10.3389/fgene.2013.00283 24339831 PMC3857533

[16] Guo JU, Agarwal V, Guo H, Bartel DP. Expanded identification and characterization of mammalian circular RNAs. Genome Biol. 2014; 15:409. 10.1186/s13059-014-0409-z 25070500 PMC4165365

[17] Kumar L, Shamsuzzama, Jadiya P, Haque R, Shukla S, Nazir A. Functional characterization of novel circular RNA molecule, circzip-2 and its synthesizing gene zip-2 in C. elegans model of Parkinson’s Disease. Mol Neurobiol. 2018. 10.1007/s12035-018-0903-5 29363043

[18] Lukiw WJ. Circular RNA (circRNA) in Alzheimer’s disease (AD). Front Genet. 2013; 4:307. 10.3389/fgene.2013.00307 24427167 PMC3875874

[19] Hansen TB, Kjems J, Damgaard CK. Circular RNA and miR-7 in cancer. Cancer Res. 2013; 73:5609–12. 10.1158/0008-5472.CAN-13-1568 24014594

[20] McMillan KJ, Murray TK, Bengoa-Vergniory N, Cordero-Llana O, Cooper J, Buckley A, Wade-Martins R, Uney JB, O’Neill MJ, Wong LF, Caldwell MA. Loss of MicroRNA-7 regulation leads to alpha-synuclein accumulation and dopaminergic neuronal loss in vivo. Mol Ther. 2017; 25:2404–14. 10.1016/j.ymthe.2017.08.017 28927576 PMC5628933

[21] Tarale P, Daiwile AP, Sivanesan S, Stöger R, Bafana A, Naoghare PK, Parmar D, Chakrabarti T, Krishnamurthi K. Manganese exposure: linking down-regulation of miRNA-7 and miRNA-433 with α-synuclein overexpression and risk of idiopathic Parkinson’s disease. Toxicol In Vitro. 2018; 46:94–101. 10.1016/j.tiv.2017.10.003 28986288

[22] Zhou Y, Lu M, Du RH, Qiao C, Jiang CY, Zhang KZ, Ding JH, Hu G. MicroRNA-7 targets Nod-like receptor protein 3 inflammasome to modulate neuroinflammation in the pathogenesis of Parkinson’s disease. Mol Neurodegener. 2016; 11:28. 10.1186/s13024-016-0094-3 27084336 PMC4833896

[23] Li S, Lv X, Zhai K, Xu R, Zhang Y, Zhao S, Qin X, Yin L, Lou J. MicroRNA-7 inhibits neuronal apoptosis in a cellular Parkinson’s disease model by targeting Bax and Sirt2. Am J Transl Res. 2016; 8:993–1004. 27158385 PMC4846942

[24] Xu J, Kao SY, Lee FJ, Song W, Jin LW, Yankner BA. Dopamine-dependent neurotoxicity of alpha-synuclein: a mechanism for selective neurodegeneration in Parkinson disease. Nat Med. 2002; 8:600–06. 10.1038/nm0602-600 12042811

[25] Musgrove RE, King AE, Dickson TC. α-Synuclein protects neurons from apoptosis downstream of free-radical production through modulation of the MAPK signalling pathway. Neurotox Res. 2013; 23:358–69. 10.1007/s12640-012-9352-5 22936601

[26] Jiang P, Gan M, Yen SH, McLean PJ, Dickson DW. Histones facilitate α-synuclein aggregation during neuronal apoptosis. Acta Neuropathol. 2017; 133:547–58. 10.1007/s00401-016-1660-z 28004278 PMC5350017

[27] Ibáñez P, Bonnet AM, Débarges B, Lohmann E, Tison F, Pollak P, Agid Y, Dürr A, Brice A. Causal relation between alpha-synuclein gene duplication and familial Parkinson’s disease. Lancet. 2004; 364:1169–71. 10.1016/S0140-6736(04)17104-3 15451225

[28] Lehri-Boufala S, Ouidja MO, Barbier-Chassefière V, Hénault E, Raisman-Vozari R, Garrigue-Antar L, Papy-Garcia D, Morin C. New roles of glycosaminoglycans in α-synuclein aggregation in a cellular model of Parkinson disease. PLoS One. 2015; 10:e0116641. 10.1371/journal.pone.0116641 25617759 PMC4305359

[29] Lenart J, Zieminska E, Diamandakis D, Lazarewicz JW. Altered expression of genes involved in programmed cell death in primary cultured rat cerebellar granule cells acutely challenged with tetrabromobisphenol A. Neurotoxicology. 2017; 63:126–36. 10.1016/j.neuro.2017.09.014 28970181

[30] Decressac M, Mattsson B, Weikop P, Lundblad M, Jakobsson J, Björklund A. TFEB-mediated autophagy rescues midbrain dopamine neurons from α-synuclein toxicity. Proc Natl Acad Sci USA. 2013; 110:E1817–26. 10.1073/pnas.1305623110 23610405 PMC3651458

